# Autonomic Imbalance and Elevated Inflammatory Cytokines in Long COVID: A Cross-Sectional Study

**DOI:** 10.7759/cureus.66971

**Published:** 2024-08-15

**Authors:** Lakshmi Jatiya, Jothi Marie Feula, Latha R, Vidhyalakshmi R, James Rajesh

**Affiliations:** 1 Department of Physiology, Aarupadai Veedu Medical College and Hospital, Vinayaka Mission's Research Foundation (VMRF), Puducherry, IND; 2 Department of Physiology, All India Institute of Medical Sciences Madurai, Madurai, IND; 3 Department of Microbiology, Aarupadai Veedu Medical College and Hospital, Vinayaka Mission's Research Foundation (VMRF), Puducherry, IND; 4 Department of Physiology, Chettinad Medical College and Hospital, Chennai, IND; 5 Forensic Medicine and Toxicology, Velammal Medical College Hospital and Research Institute, Madurai, IND

**Keywords:** autonomic imbalance, interleukin – 6, long haul covid, post covid syndrome, chronic covid, long covid

## Abstract

Introduction: Following an infection with SARS-CoV-2, the virus that causes coronavirus disease 2019 (COVID-19), many individuals fully recover. On the other hand, a few have symptoms that last for weeks, months, or even years after their initial diagnosis. Symptoms of COVID-19 persisting for four weeks and more are termed long COVID.

Aim: To assess the long-term cardiovascular morbidity by battery of cardiac autonomic function tests as well as the persistence of inflammation in COVID-recovered patients three months after initial infection.

Methodology: 150 patients were selected who had recovered from COVID-19 at least three months prior to the study. After obtaining informed written consent, a throat swab was tested for COVID-19, and those with negative reverse transcription polymerase chain reaction (RT-PCR) results were subjected to autonomic function testing. Serum interleukin-6 and C-reactive protein levels were determined by enzyme-linked immunosorbent assay (ELISA) test.

Results: Out of 150 subjects 36 were found to have autonomic dysfunction graded according to Ewing’s criteria. Individuals with autonomic dysfunction also had significantly increased inflammatory biomarker levels. There was also significant correlation between inflammatory markers and autonomic function test and heart rate variability parameters.

Conclusion: Even years after the COVID-19 pandemic was declared, new symptom patterns and syndromes such as 'long COVID' are appearing. A better understanding of the pathophysiological mechanisms of post-COVID manifestations that affect the autonomic nervous system, as well as customized therapeutic care, should help reduce COVID-19 sequelae, particularly if we act early in the disease.

## Introduction

The coronavirus disease 2019 (COVID-19) pandemic, caused by the severe acute respiratory syndrome coronavirus 2 (SARS-CoV-2), has had a devastating impact on the global population's health and economy. More than 430 million confirmed cases of COVID-19 and more than 5.9 million deaths had been reported to the World Health Organization (WHO) by February 2022 [1]. Around 80% of COVID-19 patients suffer from mild illness and recover in two to four weeks. Approximately 20% of patients end up with moderate and severe forms of illness and are hospitalised. The course of the disease is determined by proinflammatory cytokines such as interleukin-6 (IL-6) and C-reactive protein (CRP), as well as the existence of co-morbidities such as cardiovascular, respiratory, metabolic, and cancer. The question that emerges is what pathological similarities these disorders share. The reality is that all of these illnesses have an autonomic imbalance characterized by parasympathetic withdrawal and sympathetic overactivity [2].

Acute COVID is defined as signs and symptoms of COVID that last for less than four weeks. Ongoing symptomatic COVID is defined as signs and symptoms that persist after four weeks and up to 12 weeks of initial infection. Post-COVID syndrome is defined as symptoms that persist for more than 12 weeks after ruling out reinfection. Prolonged COVID, also known as Long COVID, refers to both ongoing symptomatic COVID and chronic COVID [1]. Post-COVID symptoms are quite diverse, affecting and involving several systems. Numerous pathophysiological mechanisms like direct or indirect invasion of virus, immune dysregulation of the immune system, hormonal disruptions, elevated cytokine levels as a result of immune response leading to chronic inflammation, direct tissue damage, and persistent low-grade infection have been proposed for the COVID-related symptoms [3,4].

Because COVID-19 is a new disease, much about the clinical course, in particular, the possible long-term health consequences, remains a mystery. So, in the current study we aimed to assess the long-term cardiovascular morbidity by a battery of cardiac autonomic function tests as well as the persistence of inflammation in COVID-recovered patients three months after initial infection and we also aimed to correlate the severity of autonomic dysfunction and inflammation in individuals with autonomic dysfunction.

## Materials and methods

It was a cross-sectional study conducted in the Department of Physiology, in collaboration with the Department of Pulmonary Medicine and the Department of Microbiology. Aarupadai Veedu Medical College (AVMC) Institutional Ethics Committee issued approval AV/IEC/2021/016. Study participants were 150 COVID-recovered patients after three months of infection and of age group 18 - 45 years. Based on the prevalence of long term respiratory dysfunction after three to four months of initial infection, sample size was calculated as 150, with percentage frequency of 63% and 80% confidence level [5]. Individuals with positive reverse transcription polymerase chain reaction (RT-PCR) testing for COVID-19, with preexisting cardiovascular, respiratory, endocrine and metabolic disorders, individuals on medications that affect autonomic nervous system, individuals with any acute infections, pregnant women, lactating mothers were excluded from the study. After obtaining Institutional research committee and ethical committee approval, subjects were recruited by contacting them from hospital records and also directly recruited from the pulmonology outpatient department during their follow up visits. Staff from our institute who fulfilled the inclusion criteria were also recruited for the study. Subjects were asked to report to the Department of Physiology at any time of the day. Informed written consent was obtained and throat swab was collected under sterile aseptic precautions. After negative RT-PCR testing for COVID-19 and recording of vital signs, subjects who met the inclusion criteria were recruited into the study and anthropometric parameters were recorded. Following which they were asked to report to the autonomic function test lab on the next day between 9.00 am to 10.00 am for autonomic function testing. 2 mL of venous blood was drawn for estimating serum inflammatory markers like interleukin-6 and C- reactive protein. After five minutes of supine rest heart rate variability and a battery of cardiac autonomic function tests were recorded using BIOPAC (Goleta, CA, USA) following Taskforce guidelines. The battery of autonomic function tests included blood pressure response and heart rate response to standing (30:15 ratio), heart rate variations during deep breathing (Expiration: Inspiration ratio), heart rate response during Valsalva manoeuvre, blood pressure response to sustained isometric handgrip (Δ DBP IHG). The test results were categorised as normal, early, definitive and severe autonomic imbalance as per Ewing’s criteria [6,7]. The estimation of C-reactive protein was done by immunoturbidimetry technique and Interleukin-6 was measured by chemiluminescent immunoassay (CLIA) technique.

Data were analysed using SPSS software (IBM Corp., Armonk, NY, USA). Normality of the data was tested by Kolmogorov Smirnov test. Continuous variables were expressed as mean with standard deviation. Prevalence of long-term cardiovascular morbidity and persistent inflammation were expressed as percentage and frequency.

Inflammatory marker levels across different severity grades of autonomic dysfunction were compared by one-way ANOVA. The autonomic function test parameters and inflammatory markers were correlated using Pearson’s test. P values less than 0.05 was considered statistically significant.

## Results

The demographic data of all the study subjects (n = 150) and also of subjects with autonomic dysfunction (n = 36) are mentioned in separate columns in Table 1. Out of a total of 150 participants, 36 patients had abnormal cardiac autonomic function tests. This denotes that the prevalence of autonomic dysfunction in COVID-19 is 22%. Table 2 shows the frequency of various grades of severity of autonomic dysfunction and serum levels of IL-6 and CRP in different severity groups.

**Table 1:** Demographic data of the study subjects

S.No	Parameter	Total study subjects (N = 150)	Subjects with autonomic dysfunction (N = 36)
1	Mean age in years	30.62 ± 7.3	32.17 ± 8.2
2	Male N (%)	69 (46%)	16 (44.5 %)
3	Female N (%)	81 (54%)	20 (55.5%)

**Table 2:** Frequency of severity grading of autonomic dysfunction IL-6 – Interleukin- 6, CRP – C Reactive Protein Reference range: IL-6 - <4.40 pg/mL; CRP - < 5 mg/L

Severity Grading	Number of subjects (N=150)	Frequency (%)	IL – 6 (Mean ± SD)	CRP (Mean ± SD)
Normal	114	76 %	3± 0.57 pg/mL	2.6 ± 1.1 mg/L
Early	23	15 %	5.4 ± 1.1 pg/mL	5.6 ±1.1 mg/L
Definite	13	9 %	7.7 ± 1.8 pg/mL	9.4 ± 3.6 mg/L
Severe	0	0	NA	NA

On comparison of serum inflammatory markers among the different severity grades, there were significant difference in serum inflammatory marker levels between normal autonomic function group and the groups with early and definitive autonomic dysfunction group (p < 0.001) (Table 3).

**Table 3:** Comparison of Interleukin-6 and C-Reactive Protein levels in various severity grades of autonomic imbalance Analysed by One way ANOVA. * P values less than 0.05 are considered statistically significant. IL-6 – Interleukin-6, CRP – C Reactive Protein

Inflammatory markers	Severity Grade (I)	Severity Grade (J)	Mean Difference (I-J)	Std. Error	P value	
						IL6	Normal	Early	-2.36*	.19	<0.001*	
Definitive	-4.7*	.24	<0.001*				
CRP	Normal	Early	-3.01*	.36	<0.001*	
		Definitive	-6.80*	.44	<0.001*	

On correlation of heart rate variability parameters and serum inflammatory markers (Table 4), there was significant negative correlation between root mean square of successive differences between normal heartbeats (RMSSD) (p=0.001), standard deviation of the N-N intervals (SDNN) (p=0.003) and high frequency (HF) (p=0.003). There was also significant positive correlation between serum inflammatory markers with low frequency (LF) (p=0.002) and LF/HF ratio (<0.001). On correlation of serum inflammatory markers and autonomic function parameters there was significant correlation of inflammatory markers with E/I ratio (ratio of the maximum RR interval during expiration to the minimum RR interval during inspiration following deep breathing) (p=0.002), Valsalva ratio (p=0.021) and blood pressure response to isometric hand grip (p=0.002).

**Table 4:** The correlation of heart rate variability and autonomic function test parameters and inflammatory markers in individuals with autonomic dysfunction (n=36) Analysed by Pearson correlation test. *P values less than 0.05 are considered statistically significant. LF - low frequency;  HF - high frequency; LF/HF - ratio of low frequency to high frequency; SDNN - standard deviation of the averages of the NN intervals in all 5 min segments; RMSSD - Square root of the mean squared differences of successive normal to normal intervals; 30:15 - ratio of the maximum RR interval at 30th beat to the minimum RR interval at 15th beat from supine to standing; E:I - ratio of the maximum RR interval during expiration to the minimum RR interval during inspiration following deep breathing; Δ DBP IHG - maximum rise in diastolic blood pressure above baseline following sustained handgrip.

S.NO	Parameters	Interleukin -6		C Reactive Protein	
Time domain parameters		R value	P value	R value	P value
1	RMSSD	-0.506	0.001*	-0.533	0.001*
2	SDNN	-0.393	0.015*	-0.467	0.003*
Frequency Domain parameters					
3	LF	0.52	0.001*	0.49	0.002*
4	HF	- 0.468	0.003*	- 0.465	0.003*
5	LF/HF	0.612	<0.001*	0.602	<0.001*
Cardiac autonomic function tests					
6	BP response to standing	0.190	0.25	0.38	0.179
7	30:15 ratio	- 0.19	0.11	- 0.23	0.11
8	E:I ratio	-0.33	0.039*	-0.489	0.002*
9	Valsalva Ratio	- 0.312	0.05	- 0.373	0.021*
10	BP response to isometric hand grip (Δ DBP _IHG _).	0.495	0.002*	0.485	0.002*

## Discussion

After three months of infection, 36 of the 150 COVID-19-recovered participants were found to have autonomic dysfunction. Out of these 36 with autonomic dysfunction 23 individuals had early autonomic dysfunction, 13 had definitive autonomic dysfunction and none of the subjects had severe dysfunction as per Ewing’s criteria. These individuals also experienced symptoms such as dizziness, generalized fatigue, palpitations, and frequent diarrhoea. ‘Chronic/long COVID’ describes symptoms lasting more than 12 weeks. All our study subjects were reported to have a milder form of initial COVID-19 infection and they recovered on outpatient treatment. Our findings were consistent with the findings of Torre et al., who reported 25% dysautonomia in post-COVID patients [1].

Huang et al. published the first epidemiological and clinical characteristics of COVID-19 patients (191 patients) in early March 2020, reporting that 48% of the patients confirmed with COVID-19 had significant comorbidities such as hypertension (30%), diabetes (19%), and heart disease (8%) [8]. All of the conditions listed above have been linked to increased sympathetic nervous system activity, which in many cases contributes to disease progression. These disease conditions may predispose to more sympathetic activity following COVID-19 infection. As a result, it has been postulated that this common feature may be one major factor contributing to increased morbidity and mortality in long COVID [9]. In our study we have excluded individuals with preexisting diseases. It has also been documented from our study that a discernible disruption in autonomic function persists even beyond resolution of the underlying infection. Therefore we propose that the only possible etiology of long COVID syndrome is this autonomic dysfunction. There are also similar studies in the literature investigating the possible role of autonomic dysfunction in long COVID [10-12].

In our study we also reported that our subjects had significantly elevated serum inflammatory markers. Our findings are comparable with those of Doykov et al., who found significantly elevated inflammatory biomarkers after 40 days of infection [13]. In our study, as shown in Table 4, we found a correlation between serum inflammatory markers and heart rate variability and autonomic function test parameters in participants with dysautonomia, indicating sympathetic predominance in long COVID syndrome. This finding is also consistent with the study reports explaining the interplay between inflammation and autonomic imbalance [14].

Under normal conditions with a fair sympatho-vagal balance, in reaction to inflammation, the vagal anti-inflammatory reflex activates a vagal cholinergic efferent arm. When the vagal anti-inflammatory reflex is activated, macrophages of the mononuclear phagocytic system, including resident and circulating macrophages, block cytokine production. These changes can be seen in a variety of tissues, including the lungs, heart, and brain. Considering the presence of reciprocal inhibition in the autonomic nervous system, sympatho-excitation appears to be intimately related to parasympathetic (vagal) withdrawal [1,9,14]. As a result, it is reasonable to anticipate a decrease in the neuro-vagal inflammatory reaction, which could contribute to the loss of normal inflammatory process restraint leading to long COVID. This effect was also observed in COVID-19 patients, where the cytokine storm is thought to contribute to a rapid transition from a compensated to a decompensated condition needing supplemental oxygen and/or mechanical ventilation [15].

COVID-19 patients frequently experience symptoms related to autonomic dysfunction. These symptoms have a significant impact on both the short and medium to long-term quality of life. A deeper knowledge of the pathophysiological mechanisms of post-COVID manifestations that influence the autonomic nervous system, as well as tailored therapeutic care, could help diminish COVID-19 sequelae, especially if we act in the early stages of the disease.

The study's limitations are that we recruited patients three months after their initial infection; we did not follow them up over an extended period of time. Regularly adhering to them would have provided us with a more comprehensive understanding of disease trajectory and eventual consequences.

## Conclusions

Even years after the COVID-19 pandemic was declared, new symptom patterns and syndromes such as 'long COVID' are appearing. These patterns could be explained by direct virus-induced or inflammation-mediated autonomic instability. We estimate that these disorders will account for a significant part of primary and secondary care encounters. Clinicians must understand that fast and accurate diagnosis, as well as attentive management, are critical for recovery. Prescribing non-medical therapies like yoga which reduces the sympathetic activity and increases parasympathetic activity as adjuvant with the medical therapy can accentuate the recovery and also reduced the long term sequelae.
